# Adjuvant Chemotherapy Associated with Survival Benefit Following Neoadjuvant Chemotherapy and Pancreatectomy for Pancreatic Ductal Adenocarcinoma: A Population-Based Cohort Study

**DOI:** 10.1245/s10434-021-09823-0

**Published:** 2021-03-30

**Authors:** Sivesh K. Kamarajah, Steven A. White, Samer A. Naffouje, George I Salti, Fadi Dahdaleh

**Affiliations:** 1 Department of HPB and Transplant Surgery, The Freeman Hospital, Newcastle upon Tyne, Tyne and Wear UK; 2 Institute of Cellular Medicine, Newcastle University, Newcastle Upon Tyne, Newcastle UK; 3 Department of Surgery, Queen Elizabeth Hospital Birmingham, University Hospital Birmingham NHS Trust, Birmingham, UK; 4 Department of Surgical Oncology, H. Lee Moffitt Cancer and Research Institute, Tampa, FL USA; 5 Department of General Surgery, University of Illinois Hospital and Health Sciences System, Chicago, IL USA; 6 Department of Surgical Oncology, Edward-Elmhurst Health, Naperville, IL USA

## Abstract

**Background:**

Data supporting the routine use of adjuvant chemotherapy (AC) compared with no AC (noAC) following neoadjuvant chemotherapy (NAC) and resection for pancreatic ductal adenocarcinoma (PDAC) are lacking. This study aimed to determine whether AC improves long-term survival in patients receiving NAC and resection.

**Methods:**

Patients receiving resection for PDAC following NAC from 2004 to 2016 were identified from the National Cancer Data Base (NCDB). Patients with a survival rate of < 6 months were excluded to account for immortal time bias. Propensity score matching (PSM) and Cox regression analysis were performed to account for selection bias and analyze the impact of AC on overall survival.

**Results:**

Of 4449 (68%) noAC patients and 2111 (32%) AC patients, 2016 noAC patients and 2016 AC patients remained after PSM. After matching, AC was associated with improved survival (median 29.4 vs. 24.9 months; *p* < 0.001), which remained after multivariable adjustment (HR 0.81, 95% confidence interval [CI] 0.75–0.88; *p* < 0.001). On multivariable interaction analyses, this benefit persisted irrespective of nodal status: N0 (hazard ratio [HR] 0.80, 95% CI 0.72–0.90; *p* < 0.001), N1 (HR 0.76, 95% CI 0.67–0.86; *p* < 0.001), R0 margin status (HR 0.82, 95% CI 0.75–0.89; *p* < 0.001), R1 margin status (HR 0.77, 95% CI 0.64–0.93; *p* = 0.007), no neoadjuvant radiotherapy (NART; HR 0.84, 95% CI 0.74–0.96; *p* = 0.009), and use of NART (HR 0.80, 95% CI 0.73–0.88; *p* < 0.001). Stratified analysis by nodal, margin, and NART status demonstrated consistent results.

**Conclusion:**

AC following NAC and resection is associated with improved survival, even in margin-negative and node-negative disease. These findings suggest completing planned systemic treatment should be considered in all resected PDACs previously treated with NAC.

**Supplementary Information:**

The online version contains supplementary material available at 10.1245/s10434-021-09823-0.

Multimodal therapy with pancreatectomy and adjuvant chemotherapy (AC) or chemoradiotherapy^1^–^3^ remains the standard treatment for localized pancreatic ductal adenocarcinoma (PDAC). Recently, neoadjuvant therapy (NAT) for PDAC is recognized as an acceptable treatment strategy for patients with resectable, borderline resectable, and locally advanced disease.^4^–^7^ Neoadjuvant chemotherapy (NAC) and/or neoadjuvant radiotherapy (NART) help to downstage the size and anatomic extent of the primary tumor, to improve the likelihood of a margin-negative (R0) resection, and improve the selection of patients with biologically aggressive cancers who are less likely to benefit from surgery.^8^^,^^9^ Furthermore, NAT may also maximize the number of patients receiving multimodal therapy, as up to 40% of patients do not receive AC after pancreatectomy.^8^^,^^9^ Indeed, administering all planned systemic therapy preoperatively may obviate the need for additional AC following resection, but accumulated toxicity from NAT may theoretically prohibit curative-intent surgery. Therefore, most NAT protocols include only a portion of total planned cycles to be administered upfront.^10^–^12^

Although the benefit of AC to patients who have undergone resection first is clear, the benefit of AC after NAC and resection is less well-established.^13^–^19^ First, previous retrospective analyses investigating the added benefit of AC after NAT and resection have yielded conflicting results, as some suggested benefit in certain subgroups only.^13^–^19^ Second, while guidelines from the American Society of Clinical Oncology (ASCO) and National Comprehensive Cancer Network (NCCN) recommend considering additional AC in this setting, those recommendations likely represent an extrapolation from data of adjuvant trials.^4^^,^^5^ Therefore, more high-quality data are needed to truly understand the impact of AC following NAT in patients undergoing resection for PDAC.

We sought to add evidence to this debate by performing a large, nationwide retrospective study to assess the potential benefit of AC after NAC and resection of PDAC. With contemporary data from the National Cancer Data Base (NCDB), we analyzed the association of AC with survival after resection of PDAC in patients surviving > 6 months to account for immortal time bias. We also used propensity score matching (PSM) to address treatment selection bias and assessed survival in clinically relevant subgroups of patients based on nodal and margin status.

## Methods

### Data Source

The NCDB is a joint project of the Commission on Cancer (CoC) of the American College of Surgeons and the American Cancer Society.^20^^,^^21^ Data from over 1500 CoC-accredited hospitals are gathered to include > 70% of all newly diagnosed cancers in the US. Details on demographics, facility type and location, clinicopathologic characteristics, treatment, and outcomes are available.

### Study Population

NCDB was used to identify all patients > 18 years of age diagnosed with non-metastatic PDAC undergoing resection [i.e. pancreaticoduodenectomy (PD) or distal pancreatectomy (DP)] with curative intent between 2004 and 2016. The International Classification of Diseases for Oncology, Third Edition (ICD-O-3) was used to select adenocarcinoma and to exclude other histologies (ICD-O-3 morphology codes 8240–8248). Patients with concomitant cancer diagnoses and those with missing data on receipt of perioperative chemotherapy were excluded. Patients with survival > 6 months were only included to account for immortal time bias in patients who were not able to complete AC.^22^

Center volume is defined as the annual resection volume and was divided into five quintiles (i.e. quintiles 1–5). The following patient-level characteristics were analyzed: age (36–50, 51–65, 66–80, > 80 years), race (White, Black, other), Charlson–Deyo comorbidity score (CDCC), year of diagnosis, insurance status (Medicaid/Medicare, private insurance, uninsured), zip code-level education status (i.e. < 7.0%, 7.0–12.9%, 13.0–20.9%, > 21.0%), nodal status (N0, N1, N2), tumor grade/differentiation (well, moderate, poor, anaplastic), and lymphovascular invasion (absent, present). The 8th edition of the American Joint Committee on Cancer (AJCC) staging system was used both T and N classifications. Finally, we analyzed the rates of receipt of AC as the primary exposure variable.

### Statistical Analysis

Categorical variables were compared using the Chi-square test, and non-normally distributed data were analyzed using the Mann–Whitney U test. Survival was estimated using Kaplan–Meier survival curves and compared using the log-rank test. Multivariable analyses used Cox proportional hazards models. The conditional probability of receiving AC, i.e. the propensity score, was estimated using a multivariable logistic regression model including all variables listed above. We then created balanced cohorts using 1-to-1 nearest-neighbor PSM without replacement (caliper width 0.1 standard deviations).^23^ Balance diagnostics were conducted using standardized mean differences, with a value < 0.1 indicating good balance.^23^ Sensitivity and interaction analyses were performed by nodal status (i.e. N0, N1, N2/3), margin status (i.e. R0, R1), and receipt of NART on long-term survival. A *p*-value of < 0.05 was considered statistically significant. Data analysis was performed using R Foundation statistical software (R 3.2.2) with TableOne, ggplot2, Hmisc, Matchit, and survival packages (The R Foundation for Statistical Computing, Vienna, Austria) as previously described.^24^^,^^25^ This study was exempt from Institutional Review Board approval.

## Results

### Clinicopathologic Characteristics and Propensity Score Matching

This study included 6560 patients undergoing surgical resection following NAC for PDAC, of whom 2111 (32%) received AC and 4449 (68%) did not. Baseline characteristics of the entire unmatched cohort are presented in Table 1. Baseline demographics of the unmatched cohort revealed that patients receiving AC were younger and had lower CDCC scores (Table 1). Moreover, AC patients more often had advanced tumor stages and positive lymph nodes. Of patients receiving AC, 60% (1266/2111) had also received NART, compared with 65% in no AC (noAC) patients (*p* < 0.001). To account for potential treatment selection bias, PSM was performed as described above, which resulted in well-balanced cohorts (Table 1). Standardized mean differences were calculated for each variable and ranged between 0.01 and 0.05, indicating good balance.

**Table 1:** Clinicopathologic characteristics by receipt of adjuvant chemotherapy following neoadjuvant chemotherapy and resection of pancreatic ductal adenocarcinoma in unmatched and matched cohorts

	Unmatched cohort			Matched cohort		
	noAC [*n* = 4449]	AC [*n* = 2111]	*p* value	noAC [*n* = 2061]	AC [*n* = 2061]	*p* value
Center volume						
1 (lowest)	431 (9.7)	290 (13.7)	< 0.001	277 (13.4)	277 (13.4)	1.0
2	527 (11.8)	278 (13.2)		262 (12.7)	271 (13.1)	
3	704 (15.8)	268 (12.7)		259 (12.6)	264 (12.8)	
4	1257 (28.3)	580 (27.5)		565 (27.4)	571 (27.7)	
5 (highest)	1530 (34.4)	695 (32.9)		698 (33.9)	678 (32.9)	
Facility type						
Community	906 (20.4)	559 (26.5)	< 0.001	544 (26.4)	539 (26.2)	0.8
Academic	2941 (66.1)	1354 (64.1)		1336 (64.8)	1328 (64.4)	
Others	602 (13.5)	198 (9.4)		181 (8.8)	194 (9.4)	
Facility location						
Northeast	1032 (23.2)	592 (28.0)	< 0.001	560 (27.2)	574 (27.9)	0.9
South	1598 (35.9)	628 (29.7)		639 (31.0)	620 (30.1)	
Midwest	1240 (27.9)	582 (27.6)		552 (26.8)	571 (27.7)	
West	523 (11.8)	291 (13.8)		295 (14.3)	280 (13.6)	
Unknown	56 (1.3)	18 (0.9)		15 (0.7)	16 (0.8)	
Hospital distance, miles						
< 12.5	1536 (34.5)	772 (36.6)	< 0.001	749 (36.3)	754 (36.6)	1.0
12.5–49.9	1517 (34.1)	788 (37.3)		764 (37.1)	767 (37.2)	
≥ 50 miles	1396 (31.4)	551 (26.1)		548 (26.6)	540 (26.2)	
Year of diagnosis						
2004–2005	214 (4.8)	27 (1.3)	< 0.001	29 (1.4)	27 (1.3)	1.0
2006–2007	240 (5.4)	94 (4.5)		92 (4.5)	94 (4.6)	
2008–2009	500 (11.2)	226 (10.7)		213 (10.3)	221 (10.7)	
2010–2011	811 (18.2)	421 (19.9)		396 (19.2)	409 (19.8)	
2012–2013	1086 (24.4)	529 (25.1)		517 (25.1)	516 (25.0)	
2014–2016	1598 (35.9)	814 (38.6)		814 (39.5)	794 (38.5)	
Age at diagnosis, years						
18–35	30 (0.7)	9 (0.4)	0.4	8 (0.4)	9 (0.4)	1.0
36–50	412 (9.3)	203 (9.6)		201 (9.8)	194 (9.4)	
51–65	1942 (43.7)	961 (45.6)		939 (45.6)	935 (45.4)	
66–80	1898 (42.7)	864 (41.0)		834 (40.5)	851 (41.3)	
80+	163 (3.7)	72 (3.4)		79 (3.8)	72 (3.5)	
Sex						
Male	2236 (50.3)	1102 (52.2)	0.1	1073 (52.1)	1079 (52.4)	0.9
Female	2213 (49.7)	1009 (47.8)		988 (47.9)	982 (47.6)	
Race						
White	3819 (85.8)	1905 (90.2)	< 0.001	1860 (90.2)	1857 (90.1)	1.0
Black	447 (10.0)	138 (6.5)		133 (6.5)	136 (6.6)	
Other	183 (4.1)	68 (3.2)		68 (3.3)	68 (3.3)	
CDCC score						
0–1	4136 (93.0)	1973 (93.5)	0.5	1919 (93.1)	1924 (93.4)	0.8
2+	313 (7.0)	138 (6.5)		142 (6.9)	137 (6.6)	
Insurance status						
Uninsured	87 (2.0)	32 (1.5)	< 0.001	32 (1.6)	31 (1.5)	0.8
Private insurance	1915 (43.0)	1029 (48.7)		964 (46.8)	997 (48.4)	
Medicaid	214 (4.8)	81 (3.8)		84 (4.1)	80 (3.9)	
Medicare	2022 (45.4)	924 (43.8)		929 (45.1)	910 (44.2)	
Other/unknown	211 (4.7)	45 (2.1)		52 (2.5)	43 (2.1)	
Education level, %						
> 21	570 (12.8)	233 (11.0)	0.012	220 (10.7)	226 (11.0)	0.8
13–20.9	1063 (23.9)	472 (22.4)		484 (23.5)	463 (22.5)	
7–12.9	1563 (35.1)	739 (35.0)		732 (35.5)	726 (35.2)	
< 7	1253 (28.2)	667 (31.6)		625 (30.3)	646 (31.3)	
Medical income, US%						
≤ $47,999	1704 (38.3)	712 (33.7)	0.001	707 (34.3)	699 (33.9)	0.8
$48,000–$62,999	1223 (27.5)	596 (28.2)		587 (28.5)	577 (28.0)	
$63,000+	1522 (34.2)	803 (38.0)		767 (37.2)	785 (38.1)	
Residence						
Metro	3572 (80.3)	1678 (79.5)	0.9	1628 (79.0)	1640 (79.6)	1.0
Urban	683 (15.4)	333 (15.8)		331 (16.1)	324 (15.7)	
Rural	71 (1.6)	37 (1.8)		39 (1.9)	37 (1.8)	
Unknown	123 (2.8)	63 (3.0)		63 (3.1)	60 (2.9)	
Adjuvant chemotherapy						
Single agent	1185 (26.6)	628 (29.7)	< 0.001	603 (29.3)	611 (29.6)	1.0
Multi agent	2741 (61.6)	1385 (65.6)		1359 (65.9)	1352 (65.6)	
Unknown	523 (11.8)	98 (4.6)		99 (4.8)	98 (4.8)	
Neoadjuvant radiotherapy						
None	1540 (34.6)	845 (40.0)	< 0.001	846 (41.0)	828 (40.2)	0.6
NART	2909 (65.4)	1266 (60.0)		1215 (59.0)	1233 (59.8)	
Type of surgery						
Distal pancreatectomy	3317 (74.6)	1667 (79.0)	< 0.001	1596 (77.4)	1621 (78.7)	0.4
Pancreaticoduodenectomy	1132 (25.4)	444 (21.0)		465 (22.6)	440 (21.3)	
Tumor grade						
Well	397 (8.9)	150 (7.1)	0.006	156 (7.6)	147 (7.1)	1.0
Moderate	1389 (31.2)	724 (34.3)		708 (34.4)	712 (34.5)	
Poor	874 (19.6)	435 (20.6)		424 (20.6)	429 (20.8)	
Anaplastic	1789 (40.2)	802 (38.0)		773 (37.5)	773 (37.5)	
AJCC pathological T stage						
T0	1066 (24.0)	308 (14.6)	< 0.001	289 (14.0)	278 (13.5)	1.0
T1	490 (11.0)	233 (11.0)		223 (10.8)	229 (11.1)	
T2	483 (10.9)	258 (12.2)		247 (12.0)	256 (12.4)	
T3	2288 (51.4)	1246 (59.0)		1241 (60.2)	1233 (59.8)	
T4	122 (2.7)	66 (3.1)		61 (3.0)	65 (3.2)	
AJCC pathological N stage						
N0	2866 (64.4)	1088 (51.5)	< 0.001	1057 (51.3)	1056 (51.2)	1.0
N1	1148 (25.8)	720 (34.1)		720 (34.9)	711 (34.5)	
N2	301 (6.8)	189 (9.0)		177 (8.6)	186 (9.0)	
N3	134 (3.0)	114 (5.4)		107 (5.2)	108 (5.2)	
Margin status						
Negative	3797 (85.3)	1772 (83.9)	0.1	1732 (84.0)	1729 (83.9)	0.9
Positive	652 (14.7)	339 (16.1)		329 (16.0)	332 (16.1)	
Lymphovascular invasion						
Absent	3608 (81.1)	1581 (74.9)	< 0.001	1532 (74.3)	1538 (74.6)	0.9
Present	841 (18.9)	530 (25.1)		529 (25.7)	523 (25.4)	
Length of stay						
Median (IQR)	9.0 (11.0)	8.0 (8.0)	< 0.001	9.0 (9.0)	8.0 (8.0)	0.8

Data are expressed as *n* (%)

*AC* adjuvant chemotherapy, *AJCC* American Joint Committee on Cancer, *CDCC* Charlson–Deyo comorbidity, *IQR* interquartile range, *NART* neoadjuvant radiotherapy, *noAC* no adjuvant chemotherapy

### Association of Adjuvant Chemotherapy with Survival

**Fig. 1.**
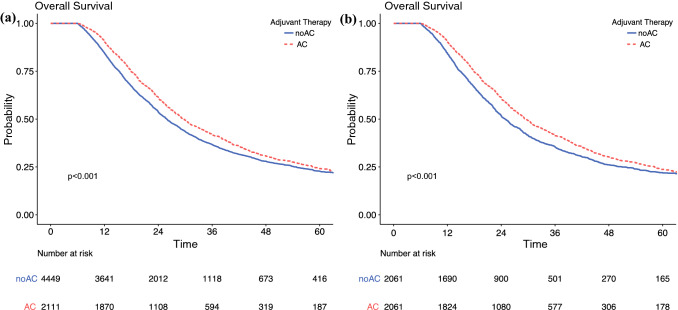
Overall survival of adjuvant chemotherapy following resection for pancreatic ductal adenocarcinoma in (**a**) unmatched and (**b**) matched cohorts. *AC* adjuvant chemotherapy, *noAC* no adjuvant chemotherapy

For the overall cohort, median survival was 27.1 months and 5-year survival was 23%. In the unmatched cohort, survival of patients receiving AC was significantly longer than those who did not receive AC (median 29.5 vs. 25.9 months; 5-year: 24% vs. 23%; *p* < 0.013) (Fig. 1, Table 2). After matching, this survival advantage persisted (median 29.4 vs. 24.9 months; 5-year: 24% vs. 20%; *p* < 0.001) (Fig. 1b, Table 2) and remained after multivariable adjustment [hazard ratio (HR) 0.81, 95% CI 0.75–0.88; *p* < 0.001] (Tables 2 and 3). On multivariable analysis and after PSM, factors associated with adverse survival included poor education level and median income, single-agent NAC, poor tumor grade, node-positive disease, margin-positive resection, and the presence of lymphovascular invasion (Table 3).

**Table 2:** Association of adjuvant chemotherapy with overall survival of patients with resected pancreatic ductal adenocarcinoma in unmatched and matched cohorts and stratified by nodal status and margin status for matched cohorts from a multivariable Cox regression model

Cohort	Chemotherapy	Median survival (IQR), months	HR (95% CI)	*p*-Value
*All patients*				
Unmatched	noAC	25.9 (25.1–26.7)	Ref	< 0.001
	AC	29.5 (28.5–30.9)	0.82 (0.76–0.87)	
Matched	noAC	24.9 (23.9–26.0)	Ref	< 0.001
	AC	29.4 (28.4–30.8)	0.81 (0.75–0.88)	
*Stratified by nodal status in the matched cohort*				
N0	noAC	29.4 (27.6–31.4)	Ref	< 0.001
	AC	34.0 (31.4–37.7)	0.80 (0.72–0.90)	
N1	noAC	22.1 (20.8–23.5)	Ref	< 0.001
	AC	28.2 (26.4–30.2)	0.76 (0.67–0.86)	
N2/3	noAC	20.0 (18.5–22.7)	Ref	0.9
	AC	21.9 (19.8–24.1)	1.01 (0.83–1.24)	
*Stratified by margin status in the matched cohort*				
R0	noAC	26.7 (25.6–28.7)	Ref	< 0.001
	AC	31.2 (29.8–34.0)	0.82 (0.75–0.89)	
R1	noAC	18.1 (16.6–19.8)	Ref	0.007
	AC	22.1 (19.6–24.0)	0.77 (0.64–0.93)	
*Stratified by neoadjuvant radiotherapy status in the matched cohort*				
No neoadjuvant radiotherapy	noAC	24.7 (22.8–27.5)	Ref	0.009
	AC	31.4 (28.6–35.5)	0.84 (0.74–0.96)	
Neoadjuvant radiotherapy	noAC	24.9 (23.8–26.2)	Ref	< 0.001
	AC	28.9 (27.4–30.5)	0.80 (0.73–0.88)	
*Stratified by surgery type*				
Pancreaticoduodenectomy	noAC	23.9 (21.5–25.7)	Ref	0.002
	AC	27.4 (25.6–30.2)	0.77 (0.66–0.91)	
Distal pancreatectomy	noAC	25.3 (24.0–26.7)	Ref	< 0.001
	AC	30.2 (28.6–31.9)	0.82 (0.75–0.89)	

*AC* adjuvant chemotherapy, *CI* confidence interval, *HR* hazard ratio, *IQR* interquartile range, *noAC* no adjuvant chemotherapy, *Ref* referent

**Table 3:** Multivariable Cox regression model of survival of patients with resected pancreatic ductal adenocarcinoma in the matched cohort

	HR (95% CI)	*p*-Value
Center volume		
Quintile 1	Ref	
Quintile 2	0.99 (0.85–1.15)	0.9
Quintile 3	0.98 (0.84–1.16)	0.8
Quintile 4	0.94 (0.80–1.11)	0.5
Quintile 5	0.78 (0.65–0.92)	0.004
Facility type		
Community	Ref	
Academic	1.05 (0.93–1.19)	0.4
Others	0.99 (0.85–1.17)	0.9
Facility location		
Northeast	Ref	
South	1.34 (1.20–1.49)	< 0.001
Midwest	1.18 (1.06–1.32)	0.003
West	1.13 (0.99–1.29)	0.1
Unknown	0.72 (0.31–1.64)	0.4
Hospital distance, miles		
< 12.5	Ref	
12.5–49.9	1.06 (0.97–1.17)	0.2
≥ 50	1.05 (0.93–1.18)	0.4
Year of diagnosis		
2004–2005	Ref	
2006–2007	0.69 (0.50–0.95)	0.025
2008–2009	0.83 (0.62–1.12)	0.2
2010–2011	0.72 (0.53–0.96)	0.027
2012–2013	0.62 (0.46–0.83)	0.001
2014–2016	0.76 (0.56–1.02)	0.1
Age at diagnosis, years		
18–35	Ref	
36–50	0.55 (0.20–1.56)	0.3
51–65	0.59 (0.21–1.70)	0.3
66–80	0.60 (0.21–1.72)	0.3
80+	0.72 (0.25–2.10)	0.5
Sex		
Male	Ref	
Female	0.97 (0.89–1.04)	0.4
Race		
White	Ref	
Black	0.90 (0.76–1.06)	0.2
Other	0.98 (0.78–1.22)	0.9
CDCC score		
0–1	Ref	
2+	1.08 (0.93–1.25)	0.3
Insurance status		
Uninsured	Ref	
Private insurance	0.80 (0.59–1.09)	0.2
Medicaid	0.77 (0.54–1.10)	0.1
Medicare	0.88 (0.64–1.21)	0.4
Unknown	0.83 (0.57–1.22)	0.4
Education level, %		
> 21%	Ref	
13–20.9	1.09 (0.94–1.26)	0.3
7–12.9	1.17 (1.01–1.36)	0.034
< 7	1.22 (1.03–1.44)	0.021
Median income, US%		
≤ $47,999	Ref	
$48,000–$62,999	0.89 (0.80–0.99)	0.037
$63,000+	0.85 (0.75–0.97)	0.012
Residence		
Metro	Ref	
Urban	0.94 (0.84–1.07)	0.4
Rural	0.73 (0.53–1.01)	0.1
Unknown	1.01 (0.80–1.27)	0.9
Neoadjuvant chemotherapy agent		
Single agent	Ref	
Multi-agent	0.85 (0.78–0.93)	< 0.001
Unknown	0.89 (0.74–1.07)	0.2
Neoadjuvant radiotherapy		
No	Ref	
Yes	1.06 (0.98–1.16)	0.2
Type of surgery		
Distal pancreatectomy	Ref	
Pancreaticoduodenectomy	1.07 (0.97–1.17)	0.2
Tumor grade		
Well	Ref	
Moderate	1.54 (1.31–1.82)	< 0.001
Poor	1.89 (1.59–2.25)	< 0.001
Anaplastic	1.43 (1.21–1.69)	< 0.001
AJCC pathological T stage		
T0	Ref	
T1	0.88 (0.74–1.05)	0.2
T2	0.92 (0.78–1.08)	0.3
T3	1.10 (0.97–1.25)	0.1
T4	1.29 (1.02–1.63)	0.036
AJCC pathological N stage		
N0	Ref	
N1	1.21 (1.10–1.32)	< 0.001
N2	1.53 (1.32–1.76)	< 0.001
N3	1.66 (1.39–1.98)	< 0.001
Margin status		
Negative	Ref	
Positive	1.56 (1.42–1.73)	< 0.001
Lymphovascular invasion		
Absent	Ref	
Present	1.12 (1.01–1.24)	0.028
Length of stay		
Mean (SD)	1.01 (1.00–1.01)	0.003
Adjuvant therapy		
None	Ref	
Yes	0.81 (0.75–0.88)	< 0.001

*AJCC* American Joint Committee on Cancer, *CDCC* Charlson–Deyo comorbidity, *CI* confidence interval, *HR* hazard ratio, *Ref* referent, *SD* standard deviation

### Interaction Between Adjuvant Chemotherapy and Nodal Status

**Fig. 2.**
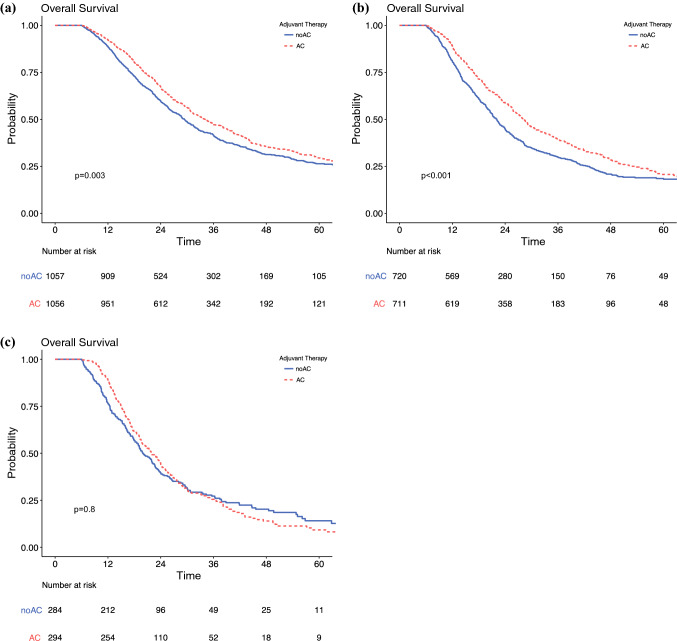
Overall survival of adjuvant chemotherapy following resection for pancreatic ductal adenocarcinoma stratified by nodal status in matched cohorts (**a**) N0, (**b**) N1, and (**c**) N2/3. *AC* adjuvant chemotherapy, *noAC* no adjuvant chemotherapy

Interaction analyses were performed to further understand the impact of AC by nodal status. In unadjusted analysis, there were significant differences in survival between AC and noAC patients in patients with N1 disease (median 39.1 vs. 34.4 months; *p* = 0.014) (Fig. 2b), but not N2/N3 disease (median 90.0 vs. 86.1 months; *p* = 0.1) (Fig. 2c). On multivariable analyses modeling the interaction between receipt of AC and nodal status, a survival benefit was again seen for patients with N0 and N1 disease but not N2/N3 disease (Table 4, electronic supplementary Table 2). As a sensitivity analysis, three separate multivariable analyses in cohorts including only those with N0, N1, and N2/N3 disease were conducted, respectively. These analyses confirmed the same findings (Table 3).

**Table 4:** Multivariable Cox regression model of survival of patients with resected pancreatic ductal adenocarcinoma in the matched cohort, with interactions between chemotherapy and nodal status and margin status

	HR (95% CI)	*p* Value
*Interaction by nodal status*		
Adjuvant chemotherapy * AJCC pathological N stage		
N0 + noAC	Ref	0.001
R0 + noAC	0.81 (0.68–0.97)	
R0 + AC	1.26 (1.12–1.42)	
R1 + noAC	0.76 (0.67–0.86)	
R1 + AC	1.56 (1.22–2.00)	
N2/3 + AC	0.99 (0.82–1.14)	
*Interaction by margin status*		
Adjuvant chemotherapy * margin status		
R0 + noAC	Ref	< 0.001
R0 + AC	0.83 (0.76–0.90)	
R1 + noAC	1.62 (1.41–1.86)	
R1 + AC	0.73 (0.57–0.93)	
*Interaction by neoadjuvant radiotherapy*		
Adjuvant chemotherapy * neoadjuvant radiotherapy		
noNART + noAC	Ref	< 0.001
noNART + AC	0.85 (0.75–0.96)	
NART + noAC	1.10 (0.98–1.23)	
NART + AC	0.74 (0.60–0.90)	
*Interaction by surgery type*		
Adjuvant chemotherapy * neoadjuvant radiotherapy		
DP + noAC	Ref	< 0.001
DP + AC	0.82 (0.75–0.90)	
PD + noAC	1.09 (0.96–1.23)	
PD + AC	0.77 (0.66–0.91)	

*AC* adjuvant chemotherapy, *AJCC* American Joint Committee on Cancer, *CI* confidence interval, *DP* distal pancreatectomy, *HR* hazard ratio, *NART* neoadjuvant radiotherapy, *noNART* no neoadjuvant radiotherapy, *noAC* no adjuvant chemotherapy, *PD* pancreaticoduodenectomy, *Ref* referent

### Interaction Between Adjuvant Chemotherapy and Margin Status

**Fig. 3.**
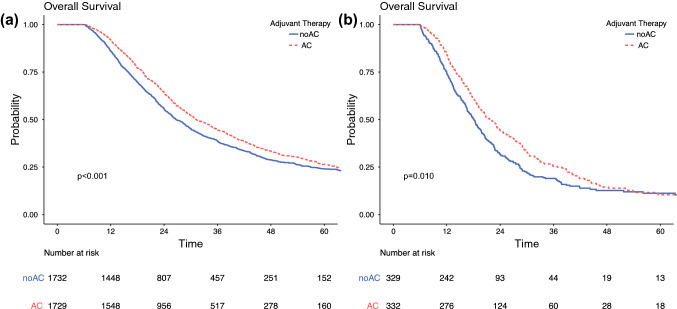
Overall survival of adjuvant chemotherapy following resection for pancreatic ductal adenocarcinoma stratified by margin status in matched cohorts (**a**) R0 and (**b**) R1. *AC* adjuvant chemotherapy, *noAC* no adjuvant chemotherapy

Interaction analyses were performed to further understand the impact of AC by margin status. In unadjusted analysis, there were significant differences in survival between AC and noAC patients in those with R0 disease (median 31.2 vs. 26.7 months; *p* < 0.001) (Fig. 3a) and in patients with R1 disease (median 22.1 vs. 18.1 months; *p* = 0.007) (Fig. 3b). On multivariable analyses modeling the interaction between receipt of AC and margin status, a survival benefit was again seen for patients with R0 margin status (HR 0.83, 95% CI 0.76–0.90; *p* < 0.001) and R1 margin status (HR 0.73, 95% CI 0.57–0.93; *p* < 0.001) (Table 4, electronic supplementary Table 3). As a sensitivity analysis, we performed two separate multivariable analyses in cohorts including only those with R0 or R1 margin, respectively. These analyses confirmed the same findings (Table 2).

### Association of Adjuvant Chemotherapy and Neoadjuvant Radiotherapy with Survival

Additional analyses were performed to further understand the impact of AC in patients who received NART. In unadjusted analysis, there were significant differences in survival between AC and noAC patients in those who did not receive NART (median 31.4 vs. 24.7 months; *p* = 0.009) (electronic supplementary Fig. 1a) and those who did (median 28.9 vs. 24.9 months; *p* < 0.001) (electronic supplementary Fig. 1b). On multivariable analyses modeling the interaction between receipt of AC and radiotherapy, a survival benefit was again seen for patients without NART (HR 0.84, 95% CI 0.74–0.96; *p* < 0.001) and those with NART (HR 0.80, 95% CI 0.73–0.88; *p* < 0.001) (electronic supplementary Table 4). As a sensitivity analysis, we performed two separate multivariable analyses in cohorts including only those without and with adjuvant radiotherapy, respectively. These analyses confirmed the same findings (Table 2).

### Association of Adjuvant Chemotherapy and Neoadjuvant Radiotherapy with Survival

Additional analyses were performed to further understand the impact of AC in patients by surgery type (i.e. PD or DP). In unadjusted analysis, there were significant differences in survival between AC and noAC patients in those who underwent PD (median 27.4 vs. 23.9 months; *p* = 0.002) and those who underwent DP (median 30.2 vs. 25.3 months; *p* < 0.001). On multivariable analyses modeling the interaction between receipt of AC and radiotherapy, a survival benefit was again seen for patients who underwent PD (HR 0.77, 95% CI 0.66–0.91; *p* < 0.001) and DP (HR 0.82, 95% CI 0.75–0.90; *p* < 0.001) (Table 4). As a sensitivity analysis, we performed two separate multivariable analyses in cohorts including only those who underwent PD and DP, respectively. These analyses confirmed the same findings (Table 2).

## Discussion

Although current national guidelines from ASCO and NCCN recommend considering additional AC in patients following NAT and surgery, no high-level data exist to support this recommendation.^4^^,^^5^ This national population-based cohort utilizing the NCDB and including 6560 patients who received NAC followed by resection for PDAC, demonstrated AC was associated with improved overall survival after accounting for potential biases through PSM. Stratified analyses by nodal and margin status demonstrated survival benefit of AC remained, even in patients considered to be at low risk of recurrence (i.e. margin-negative and node-negative disease. Furthermore, stratified analysis by receipt of NART also demonstrated a similar protective effect of AC. These findings collectively suggest that completing planned systemic therapy should be considered after NAC and surgery for PDAC, whenever possible.

To date, evidence on whether AC after NAT and surgery confers incremental advantage remains an ongoing debate as several retrospective studies on this matter have yielded conflicting results.^13^–^18^ Previous NCDB analyses by Swords et al.^13^ and de Geus et al.^14^ have attempted to study the benefit of AC following NAT. Swords et al.^13^ reported that AC was associated with improved survival only in patients with a lymph node ratio between 0.01 and 0.14, not in patients with node-negative disease or a lymph node ratio > 0.15. On the other hand, de Geus et al.^14^ found AC was not associated with improved survival in comparable settings, even in patients with node-positive or margin-positive disease. However, both studies had limitations, which were addressed in this study. First, our study utilized a contemporary edition of NCDB, which allowed for a larger cohort to be scrutinized, particularly as NAT use increased over time. For example, in the study by de Geus et al.^14^ 833 noAC and 524 AC patients were included, compared with 4449 and 2111, respectively, in this current study. This likely increased power and generalizability. Second, the present study adopted PSM to adjust for selection biases for receiving either AC or noAC to create well-balanced cohorts. Finally, previous analyses^13^^,^^14^ did not establish any survival benefit for AC in patients with low-risk disease, which was demonstrated in the present study.

Previous single-center institutional series on this topic have also been limited by very small sample sizes, precluding valid conclusions to be drawn.^15^–^18^ A recent retrospective analyses by Perri et al. that included 245 patients demonstrated noAC patients had received more chemotherapy cycles preoperatively (median 4 vs. 3), had larger tumors (2.8 vs. 2.4 cm), and had higher preoperative cancer antigen (CA) 19-9 levels (32 vs. 24 U/mL).^18^ After PSM, AC was found to be associated with improved recurrence-free survival (median 17 vs. 12 months) but not overall survival (median 42 vs. 32 months). Although the authors concluded that AC should be universally considered after NAT and surgery, subgroup analyses by nodal or margin status are lacking. In a previous study from the same group, AC was associated with improved survival only in patients with a low lymph ratio and in node-negative disease.^15^

The presence of high-risk factors, such as nodal involvement or positive margins, is commonly used to select patients for adjuvant therapy, as evidence by the distribution of AC use in the unmatched cohort. However, systemic and local recurrence in patients perceived to have low-risk disease are still as high as 40%^26^^,^^27^ and 50%, respectively.^28^^,^^29^ This suggests that clinicians may underestimate the risk of relapse in PDAC patients perceived to have favorable pathologic features after resection.^26^^,^^27^ To the authors’ knowledge, no published studies have explored the role of AC specifically in patients with margin-negative or node-negative disease. These data suggest that AC is likely beneficial in most patients and is consistent with the notion that localized PDAC should be approached as a systemic disease.^30^ While no specific criteria exist to aid in identifying patients most likely to benefit from AC, a recent study by Liu et al. found that CA19-9 response to NAT predicted incremental survival advantage, with patients only garnering benefit when levels failed to drop below 50% from baseline.^31^ While these results are important to bear in mind, this study supports considering AC universally in the absence of higher-quality data.

This study has several key strengths that should be emphasized. First, the study utilizes a large and contemporary national cohort that probably resembles real-world trends and outcomes. Indeed, NCDB is particularly suited to test this study’s stated hypothesis given an impressive level of granularity and high-quality standards. Second, detailed subgroup analyses in patients with negative lymph nodes, negative resection margins, and those who received NART were conducted to better inform decision making in clinically relevant scenarios. Lastly, potential treatment selection bias is minimized through PSM and subgroup and sensitivity analyses. We believe this lends further credibility to the stated conclusions.

Limitations of this analysis should also be acknowledged. First, this was a non-randomized, retrospective cohort study that is susceptible to bias. We attempted to minimize bias through PSM but potential unmeasured covariates may have contributed to the observed outcomes. Furthermore, patients with survival of < 6 months were excluded, primarily to account for patients who did not survive long enough to receive AC; however, it is possible that doing so also excluded patients who died due to AC-related complications, although this is likely to be a small group. Second, 60% of patients in this analysis received NART in addition to NAC. This may therefore limit generalizability as NART is uncommonly used in resectable tumors. To accommodate for that, we conducted a separate analysis according to receipt of NART and confirmed the advantage of AC in this subgroup. Third, details on AC agent(s) were limited to single- or multi-agent categories, with no further information. Nevertheless, a majority of patients in this study received multi-agent AC, which perhaps indicates that most NAC patients ultimately recover well from pancreatectomy and are eligible to receive more than single-agent regimens. Finally, it is important to note that the number of NAC cycles received is unclear from NCDB and that a recommendation for additional AC likely does not apply to patients who received longer courses of NAC, as endorsed by some investigators.^32^ Hence, this study cannot provide specific guidance with respect to the duration of either preoperative or postoperative therapy, differentiation between chemotherapy from chemoradiation therapy versus chemotherapy plus chemoradiation, the subsets of patients for whom these treatments are most beneficial, and the extent to which histopathologic analysis of the surgical specimen may inform the postoperative treatment regimen. Ongoing randomized trials will likely answer these questions.

## Conclusion

Adjuvant chemotherapy following resection is associated with improved long-term survival in patients receiving NAC, even in margin-negative and node-negative disease. These findings suggest that AC should be considered in patients who did not complete all intended systemic treatments upfront, whenever permissible.

## Supplementary Information

Below is the link to the electronic supplementary material.

Supplementary file1 (DOCX 35 KB)

Supplementary file2 (DOCX 396 KB)
