# Supplementary material for: Adjuvant Chemotherapy Associated with Survival Benefit Following Neoadjuvant Chemotherapy and Pancreatectomy for Pancreatic Ductal Adenocarcinoma: A Population-Based Cohort Study
Source: Ann Surg Oncol. 2021 Mar 30;28(11):6790–802. doi: 10.1245/s10434-021-09823-0 (PMC8460503; doi:10.1245/s10434-021-09823-0)
Supplement: Supplementary file 1 — Supplementary file1 (DOCX 35 KB) [file 10434_2021_9823_MOESM1_ESM.docx]

# Supplementary Table 1 Cox regression on overall survival for resected pancreatic ductal adenocarcinoma in unmatched cohort

|  |  | **Hazard ratio (CI_95%_)** | **p-value** |
| --- | --- | --- | --- |
| Center Volume | Quintile 1 | REF |  |
|  | Quintile 2 | 1.01 (0.89-1.15) | 0.8 |
|  | Quintile 3 | 1.01 (0.89-1.15) | 0.8 |
|  | Quintile 4 | 0.93 (0.81-1.06) | 0.3 |
|  | Quintile 5 | 0.79 (0.69-0.92) | 0.002 |
| Facility Type | Community | REF |  |
|  | Academic | 1.03 (0.93-1.14) | 0.6 |
|  | Others | 1.02 (0.91-1.15) | 0.7 |
| Facility Location | Northeast | REF |  |
|  | South | 1.33 (1.22-1.46) | <0.001 |
|  | Midwest | 1.19 (1.09-1.30) | <0.001 |
|  | West | 1.17 (1.05-1.31) | 0.005 |
|  | Unknown | 0.91 (0.53-1.54) | 0.7 |
| Hospital Distance | <12.5 miles | REF |  |
|  | 12.5-49.9 miles | 1.09 (1.01-1.18) | 0.025 |
|  | >/=50 miles | 1.06 (0.97-1.16) | 0.2 |
| Year of Diagnosis | 2004-2005 | REF |  |
|  | 2006-2007 | 0.75 (0.62-0.89) | 0.002 |
|  | 2008-2009 | 0.90 (0.76-1.05) | 0.2 |
|  | 2010-2011 | 0.79 (0.67-0.92) | 0.002 |
|  | 2012-2013 | 0.72 (0.61-0.84) | <0.001 |
|  | 2014-2016 | 0.82 (0.70-0.96) | 0.014 |
| Age at Diagnosis, years | 18-35 | REF |  |
|  | 36-50 | 0.80 (0.41-1.55) | 0.5 |
|  | 51-65 | 0.90 (0.46-1.76) | 0.8 |
|  | 66-80 | 0.94 (0.48-1.85) | 0.9 |
|  | 80+ | 1.11 (0.56-2.21) | 0.8 |
| Sex | Male | REF |  |
|  | Female | 0.98 (0.92-1.04) | 0.5 |
| Race | White | REF |  |
|  | Black | 0.92 (0.82-1.03) | 0.2 |
|  | Other | 0.85 (0.72-1.01) | 0.1 |
| CDCC Score | 0-1 | REF |  |
|  | 2+ | 1.07 (0.95-1.20) | 0.3 |
| Insurance Status | Uninsured | REF |  |
|  | Private Insurance | 1.01 (0.80-1.28) | 0.9 |
|  | Medicaid | 1.04 (0.80-1.36) | 0.8 |
|  | Medicare | 1.13 (0.89-1.43) | 0.3 |
|  | Other/Unknown | 1.11 (0.84-1.46) | 0.5 |
| Education Level | >21% | REF |  |
|  | 13%-20.9% | 1.11 (1.00-1.24) | 0.1 |
|  | 7%-12.9% | 1.13 (1.01-1.27) | 0.03 |
|  | <7% | 1.22 (1.07-1.39) | 0.002 |
| Medical Income | </=$47,999 | REF |  |
|  | $48,000-$62,999 | 0.90 (0.82-0.98) | 0.011 |
|  | $63,000 + | 0.87 (0.78-0.96) | 0.005 |
| Residence | Metro | REF |  |
|  | Urban | 0.96 (0.87-1.06) | 0.4 |
|  | Rural | 0.88 (0.69-1.14) | 0.3 |
|  | Unknown | 1.06 (0.88-1.27) | 0.6 |
| Neoadjuvant Chemotherapy Agent | Single Agent | REF |  |
|  | Multi Agent | 0.83 (0.77-0.89) | <0.001 |
|  | Unknown | 0.84 (0.75-0.94) | 0.002 |
| Neoadjuvant Radiotherapy | None | REF |  |
|  | NART | 1.06 (0.99-1.14) | 0.1 |
| Type of Surgery | Pancreaticoduodenectomy | REF |  |
|  | Distal Pancreatectomy | 0.91 (0.85-0.98) | 0.011 |
| Tumour Grade | Well | REF |  |
|  | Moderate | 1.41 (1.24-1.59) | <0.001 |
|  | Poor | 1.72 (1.51-1.95) | <0.001 |
|  | Anaplastic | 1.31 (1.15-1.48) | <0.001 |
| AJCC Pathological T Stage | T0 | REF |  |
|  | T1 | 0.90 (0.79-1.02) | 0.1 |
|  | T2 | 1.03 (0.91-1.15) | 0.7 |
|  | T3 | 1.19 (1.09-1.30) | <0.001 |
|  | T4 | 1.53 (1.27-1.83) | <0.001 |
| AJCC Pathological N Stage | N0 | REF |  |
|  | N1 | 1.18 (1.10-1.27) | <0.001 |
|  | N2 | 1.51 (1.34-1.70) | <0.001 |
|  | N3 | 1.70 (1.45-2.00) | <0.001 |
| Margin Status | Negative | REF |  |
|  | Positive | 1.53 (1.41-1.66) | <0.001 |
| Lymphovascular Invasion | Absent | REF |  |
|  | Present | 1.11 (1.02-1.21) | 0.014 |
| Length of Stay | Mean (SD) | 1.01 (1.00-1.01) | <0.001 |
| Adjuvant Therapy | None | REF |  |
|  | Yes | 0.82 (0.76-0.87) | <0.001 |

*Abbreviations: AC: adjuvant chemotherapy, CDCC: Charlson-Deyo comorbidity, CI: confidence interval* ***Additional variables included into the propensity matching omitted from tables were hospital factors (hospital distance), patient factors (race, residence)*

# Supplementary Table 3 Multivariable cox regression model of survival of patients with resected pancreatic ductal adenocarcinoma in matched cohort, with interactions between adjuvant chemotherapy and nodal status

|  |  | **Hazard ratio (CI_95%_)** | **p-value** |
| --- | --- | --- | --- |
| Center Volume | Quintile 1 | REF |  |
|  | Quintile 2 | 0.99 (0.85-1.15) | 0.9 |
|  | Quintile 3 | 0.98 (0.84-1.16) | 0.8 |
|  | Quintile 4 | 0.95 (0.81-1.11) | 0.5 |
|  | Quintile 5 | 0.78 (0.65-0.93) | 0.005 |
| Facility Type | Community | REF |  |
|  | Academic | 1.05 (0.92-1.19) | 0.5 |
|  | Others | 0.99 (0.85-1.16) | 0.9 |
| Facility Location | Northeast | REF |  |
|  | South | 1.34 (1.20-1.49) | 0.001 |
|  | Midwest | 1.19 (1.06-1.32) | 0.002 |
|  | West | 1.14 (1.00-1.30) | 0.1 |
|  | Unknown | 0.71 (0.31-1.62) | 0.4 |
| Hospital Distance | <12.5 miles | REF |  |
|  | 12.5-49.9 miles | 1.06 (0.97-1.17) | 0.2 |
|  | >/=50 miles | 1.04 (0.93-1.18) | 0.5 |
| Year of Diagnosis | 2004-2005 | REF |  |
|  | 2006-2007 | 0.69 (0.50-0.95) | 0.025 |
|  | 2008-2009 | 0.83 (0.62-1.12) | 0.2 |
|  | 2010-2011 | 0.71 (0.53-0.96) | 0.024 |
|  | 2012-2013 | 0.61 (0.46-0.82) | 0.001 |
|  | 2014-2016 | 0.76 (0.56-1.02) | 0.1 |
| Age at Diagnosis, years | 18-35 | REF |  |
|  | 36-50 | 0.54 (0.19-1.52) | 0.2 |
|  | 51-65 | 0.58 (0.20-1.66) | 0.3 |
|  | 66-80 | 0.59 (0.20-1.69) | 0.3 |
|  | 80+ | 0.70 (0.24-2.04) | 0.5 |
| Sex | Male | REF |  |
|  | Female | 0.97 (0.89-1.04) | 0.4 |
| Race | White | REF |  |
|  | Black | 0.90 (0.76-1.07) | 0.2 |
|  | Other | 0.99 (0.79-1.24) | 0.9 |
| CDCC Score | 0-1 | REF |  |
|  | 2+ | 1.08 (0.93-1.25) | 0.3 |
| Insurance Status | Uninsured | REF |  |
|  | Private Insurance | 0.80 (0.59-1.08) | 0.1 |
|  | Medicaid | 0.76 (0.53-1.09) | 0.1 |
|  | Medicare | 0.88 (0.64-1.20) | 0.4 |
|  | Unknown | 0.82 (0.56-1.21) | 0.3 |
| Education Level | >21% | REF |  |
|  | 13%-20.9% | 1.08 (0.94-1.25) | 0.3 |
|  | 7%-12.9% | 1.17 (1.01-1.36) | 0.036 |
|  | <7% | 1.22 (1.03-1.44) | 0.022 |
| Medical Income | </=$47,999 | REF |  |
|  | $48,000-$62,999 | 0.89 (0.80-0.99) | 0.037 |
|  | $63,000 + | 0.85 (0.75-0.97) | 0.014 |
| Residence | Metro | REF |  |
|  | Urban | 0.95 (0.84-1.07) | 0.4 |
|  | Rural | 0.74 (0.53-1.02) | 0.1 |
|  | Unknown | 1.01 (0.81-1.27) | 0.9 |
| Neoadjuvant Chemotherapy Agent | Single Agent | REF |  |
|  | Multi Agent | 0.85 (0.78-0.93) | <0.001 |
|  | Unknown | 0.89 (0.73-1.07) | 0.2 |
| Neoadjuvant Radiotherapy | No | REF |  |
|  | Yes | 1.07 (0.98-1.16) | 0.2 |
| Type of Surgery | Distal Pancreatectomy | REF |  |
|  | Pancreaticoduodenectomy | 1.07 (0.98-1.17) | 0.1 |
| Tumour Grade | Well | REF |  |
|  | Moderate | 1.54 (1.31-1.82) | <0.001 |
|  | Poor | 1.89 (1.59-2.25) | <0.001 |
|  | Anaplastic | 1.43 (1.21-1.69) | <0.001 |
| AJCC Pathological T Stage | T0 | REF |  |
|  | T1 | 0.88 (0.74-1.05) | 0.2 |
|  | T2 | 0.92 (0.79-1.09) | 0.3 |
|  | T3 | 1.11 (0.98-1.26) | 0.1 |
|  | T4 | 1.30 (1.03-1.65) | 0.029 |
| Margin Status | Negative | REF |  |
|  | Positive | 1.57 (1.42-1.73) | <0.001 |
| Lymphovascular Invasion | Absent | REF |  |
|  | Present | 1.12 (1.01-1.24) | 0.03 |
| Length of Stay | Mean (SD) | 1.01 (1.00-1.01) | 0.003 |
| Adjuvant Therapy * AJCC Pathological N Stage | N0 + noAC | REF |  |
|  | N0 + AC | 0.81 (0.68-0.97) | <0.001 |
|  | N1 + noAC | 1.26 (1.12-1.42) | <0.001 |
|  | N1 + AC | 0.76 (0.67-0.86) | <0.001 |
|  | N2/3 + noAC | 1.56 (1.22-2.00) | <0.001 |
|  | N2/3 + AC | 0.99 (0.82-1.14) | 0.8 |

# *Abbreviations: AC: adjuvant chemotherapy, CDCC: Charlson-Deyo comorbidity, CI: confidence interval, REF: referent **Additional variables included into the propensity matching omitted from tables were hospital factors (hospital distance), patient factors (race, education level, residence)*

# Supplementary Table 4 Multivariable Cox regression model of survival of patients with resected pancreatic ductal adenocarcinoma in matched cohort, with interactions between chemotherapy and margin status

|  |  | **Hazard ratio (CI_95%_)** | **p-value** |
| --- | --- | --- | --- |
| Center Volume | Quintile 1 | REF |  |
|  | Quintile 2 | 0.99 (0.85-1.15) | 0.9 |
|  | Quintile 3 | 0.98 (0.84-1.15) | 0.8 |
|  | Quintile 4 | 0.94 (0.80-1.10) | 0.5 |
|  | Quintile 5 | 0.77 (0.65-0.92) | 0.004 |
| Facility Type | Community | REF |  |
|  | Academic | 1.05 (0.93-1.19) | 0.4 |
|  | Others | 0.99 (0.85-1.17) | 0.9 |
| Facility Location | Northeast | REF |  |
|  | South | 1.34 (1.20-1.49) | 0.001 |
|  | Midwest | 1.18 (1.06-1.32) | 0.003 |
|  | West | 1.14 (1.00-1.30) | 0.1 |
|  | Unknown | 0.71 (0.31-1.63) | 0.4 |
| Hospital Distance | <12.5 miles | REF |  |
|  | 12.5-49.9 miles | 1.06 (0.97-1.17) | 0.2 |
|  | >/=50 miles | 1.05 (0.93-1.18) | 0.4 |
| Year of Diagnosis | 2004-2005 | REF |  |
|  | 2006-2007 | 0.70 (0.50-0.96) | 0.026 |
|  | 2008-2009 | 0.84 (0.62-1.13) | 0.2 |
|  | 2010-2011 | 0.72 (0.54-0.96) | 0.028 |
|  | 2012-2013 | 0.62 (0.46-0.83) | 0.001 |
|  | 2014-2016 | 0.76 (0.56-1.02) | 0.1 |
| Age at Diagnosis, years | 18-35 | REF |  |
|  | 36-50 | 0.55 (0.19-1.56) | 0.3 |
|  | 51-65 | 0.59 (0.21-1.69) | 0.3 |
|  | 66-80 | 0.60 (0.21-1.71) | 0.3 |
|  | 80+ | 0.72 (0.25-2.09) | 0.5 |
| Sex | Male | REF |  |
|  | Female | 0.97 (0.89-1.04) | 0.4 |
| Race | White | REF |  |
|  | Black | 0.90 (0.76-1.07) | 0.2 |
|  | Other | 0.98 (0.78-1.23) | 0.9 |
| CDCC Score | 0-1 | REF |  |
|  | 2+ | 1.08 (0.93-1.25) | 0.3 |
| Insurance Status | Uninsured | REF |  |
|  | Private Insurance | 0.80 (0.59-1.08) | 0.1 |
|  | Medicaid | 0.76 (0.53-1.09) | 0.1 |
|  | Medicare | 0.88 (0.64-1.20) | 0.4 |
|  | Unknown | 0.83 (0.56-1.22) | 0.3 |
| Education Level | >21% | REF |  |
|  | 13%-20.9% | 1.09 (0.94-1.26) | 0.3 |
|  | 7%-12.9% | 1.17 (1.01-1.36) | 0.034 |
|  | <7% | 1.22 (1.03-1.44) | 0.02 |
| Medical Income | </=$47,999 | REF |  |
|  | $48,000-$62,999 | 0.89 (0.80-0.99) | 0.037 |
|  | $63,000 + | 0.85 (0.75-0.96) | 0.012 |
| Residence | Metro | REF |  |
|  | Urban | 0.95 (0.84-1.07) | 0.4 |
|  | Rural | 0.73 (0.53-1.01) | 0.1 |
|  | Unknown | 1.01 (0.80-1.27) | 0.9 |
| Neoadjuvant Chemotherapy Agent | Single Agent | REF |  |
|  | Multi Agent | 0.85 (0.78-0.93) | 0.001 |
|  | Unknown | 0.89 (0.74-1.07) | 0.2 |
| Neoadjuvant Radiotherapy | No | REF |  |
|  | Yes | 1.07 (0.98-1.16) | 0.2 |
| Type of Surgery | Distal Pancreatectomy | REF |  |
|  | Pancreaticoduodenectomy | 1.07 (0.97-1.17) | 0.2 |
| Tumour Grade | Well | REF |  |
|  | Moderate | 1.54 (1.31-1.82) | 0.001 |
|  | Poor | 1.90 (1.59-2.26) | 0.001 |
|  | Anaplastic | 1.43 (1.21-1.69) | 0.001 |
| AJCC Pathological T Stage | T0 | REF |  |
|  | T1 | 0.88 (0.74-1.05) | 0.2 |
|  | T2 | 0.92 (0.78-1.08) | 0.3 |
|  | T3 | 1.10 (0.97-1.25) | 0.1 |
|  | T4 | 1.28 (1.01-1.62) | 0.04 |
| AJCC Pathological N Stage | N0 | REF |  |
|  | N1 | 1.21 (1.10-1.32) | 0.001 |
|  | N2 | 1.52 (1.32-1.76) | 0.001 |
|  | N3 | 1.66 (1.39-1.98) | 0.001 |
| Lymphovascular Invasion | Absent | REF |  |
|  | Present | 1.12 (1.01-1.24) | 0.03 |
| Length of Stay | Mean (SD) | 1.01 (1.00-1.01) | 0.003 |
| Adjuvant Therapy * Margin Status | R0 + noAC | REF |  |
|  | R0 + AC | 0.83 (0.76-0.90) | 0.001 |
|  | R1 + noAC | 1.62 (1.41-1.86) | <0.001 |
|  | R1 + AC | 0.73 (0.57-0.93) | 0.001 |

** Abbreviations: AC: adjuvant chemotherapy, AJCC: American Joint Commission on Cancer, CDCC: Charlson-Deyo comorbidity, CI: confidence interval, REF: referent **Additional variables included into the propensity matching omitted from tables were hospital factors (hospital distance), patient factors (race, education level, residence)*

Supplementary Table 5 Multivariable Cox regression model of survival of patients with resected pancreatic ductal adenocarcinoma in matched cohort, with interactions between chemotherapy and radiotherapy

|  |  | **Hazard ratio (CI_95%_)** | **p-value** |
| --- | --- | --- | --- |
| Center Volume | Quintile 1 | REF |  |
|  | Quintile 2 | 0.99 (0.85-1.15) | 0.9 |
|  | Quintile 3 | 0.99 (0.84-1.16) | 0.9 |
|  | Quintile 4 | 0.94 (0.80-1.11) | 0.5 |
|  | Quintile 5 | 0.78 (0.65-0.92) | 0.004 |
| Facility Type | Community | REF |  |
|  | Academic | 1.05 (0.93-1.19) | 0.4 |
|  | Others | 1.00 (0.85-1.17) | 1.0 |
| Facility Location | Northeast | REF |  |
|  | South | 1.34 (1.20-1.49) | 0.001 |
|  | Midwest | 1.18 (1.06-1.31) | 0.003 |
|  | West | 1.14 (1.00-1.30) | 0.1 |
|  | Unknown | 0.72 (0.31-1.64) | 0.4 |
| Hospital Distance | <12.5 miles | REF |  |
|  | 12.5-49.9 miles | 1.06 (0.97-1.17) | 0.2 |
|  | >/=50 miles | 1.05 (0.93-1.18) | 0.5 |
| Year of Diagnosis | 2004-2005 | REF |  |
|  | 2006-2007 | 0.70 (0.50-0.96) | 0.027 |
|  | 2008-2009 | 0.84 (0.62-1.13) | 0.2 |
|  | 2010-2011 | 0.72 (0.54-0.96) | 0.027 |
|  | 2012-2013 | 0.62 (0.46-0.83) | 0.001 |
|  | 2014-2016 | 0.76 (0.56-1.02) | 0.1 |
| Age at Diagnosis, years | 18-35 | REF |  |
|  | 36-50 | 0.55 (0.19-1.56) | 0.3 |
|  | 51-65 | 0.59 (0.21-1.69) | 0.3 |
|  | 66-80 | 0.59 (0.21-1.71) | 0.3 |
|  | 80+ | 0.72 (0.25-2.08) | 0.5 |
| Sex | Male | REF |  |
|  | Female | 0.97 (0.89-1.04) | 0.4 |
| Race | White | REF |  |
|  | Black | 0.90 (0.76-1.06) | 0.2 |
|  | Other | 0.98 (0.78-1.22) | 0.8 |
| CDCC Score | 0-1 | REF |  |
|  | 2+ | 1.08 (0.93-1.25) | 0.3 |
| Insurance Status | Uninsured | REF |  |
|  | Private Insurance | 0.80 (0.59-1.09) | 0.2 |
|  | Medicaid | 0.77 (0.54-1.10) | 0.1 |
|  | Medicare | 0.88 (0.64-1.21) | 0.4 |
|  | Unknown | 0.83 (0.57-1.22) | 0.4 |
| Education Level | >21% | REF |  |
|  | 13%-20.9% | 1.08 (0.94-1.25) | 0.3 |
|  | 7%-12.9% | 1.17 (1.01-1.36) | 0.037 |
|  | <7% | 1.21 (1.03-1.44) | 0.023 |
| Medical Income | </=$47,999 | REF |  |
|  | $48,000-$62,999 | 0.89 (0.80-0.99) | 0.039 |
|  | $63,000 + | 0.85 (0.75-0.97) | 0.013 |
| Residence | Metro | REF |  |
|  | Urban | 0.95 (0.84-1.07) | 0.4 |
|  | Rural | 0.74 (0.53-1.01) | 0.1 |
|  | Unknown | 1.01 (0.80-1.27) | 0.9 |
| Neoadjuvant Chemotherapy Agent | Single Agent | REF |  |
|  | Multi Agent | 0.85 (0.78-0.93) | 0.001 |
|  | Unknown | 0.89 (0.74-1.07) | 0.2 |
| Type of Surgery | Distal Pancreatectomy | REF |  |
|  | Pancreaticoduodenectomy | 1.07 (0.97-1.17) | 0.2 |
| Tumour Grade | Well | REF |  |
|  | Moderate | 1.55 (1.31-1.83) | 0.001 |
|  | Poor | 1.90 (1.59-2.26) | 0.001 |
|  | Anaplastic | 1.43 (1.21-1.69) | 0.001 |
| AJCC Pathological T Stage | T0 | REF |  |
|  | T1 | 0.88 (0.74-1.05) | 0.2 |
|  | T2 | 0.92 (0.78-1.08) | 0.3 |
|  | T3 | 1.10 (0.97-1.25) | 0.1 |
|  | T4 | 1.29 (1.02-1.63) | 0.036 |
| AJCC Pathological N Stage | N0 | REF |  |
|  | N1 | 1.21 (1.10-1.32) | 0.001 |
|  | N2 | 1.53 (1.32-1.76) | 0.001 |
|  | N3 | 1.66 (1.39-1.98) | 0.001 |
| Margin Status | Negative | REF |  |
|  | Positive | 1.57 (1.42-1.73) | 0.001 |
| Lymphovascular Invasion | Absent | REF |  |
|  | Present | 1.12 (1.01-1.24) | 0.028 |
| Length of Stay | Mean (SD) | 1.01 (1.00-1.01) | 0.003 |
| Adjuvant Therapy | noNART + noAC | REF |  |
|  | noNART + AC | 0.85 (0.75-0.96) | 0.009 |
| Neoadjuvant Radiotherapy | NART + noAC | 1.10 (0.98-1.23) | 0.2 |
|  | NART + AC | 0.74 (0.60-0.90) | <0.001 |

**Abbreviations: AC: adjuvant chemotherapy, AJCC: American Joint Commission on Cancer, ART: Adjuvant radiotherapy, CDCC: Charlson-Deyo comorbidity, CI: confidence interval, noAC: No adjuvant chemotherapy, REF: referent **Additional variables included into the propensity matching omitted from tables were hospital factors (hospital distance), patient factors (race, education level, residence)*
