# Supplementary material for: Adjuvant Chemotherapy Associated with Survival Benefit Following Neoadjuvant Chemotherapy and Pancreatectomy for Pancreatic Ductal Adenocarcinoma: A Population-Based Cohort Study
Source: Ann Surg Oncol. 2021 Mar 30;28(11):6790–802. doi: 10.1245/s10434-021-09823-0 (PMC8460503; doi:10.1245/s10434-021-09823-0)
Supplement: Supplementary file 2 — Supplementary file2 (DOCX 396 KB) [file 10434_2021_9823_MOESM2_ESM.docx]

Supplementary Figure 1 Overall survival of adjuvant chemotherapy following resection for pancreatic ductal adenocarcinoma stratified by receipt of neoadjuvant radiotherapy in matched cohorts (A) No neoadjuvant radiotherapy (B) Neoadjuvant radiotherapy

**A**

**B**

p=0.002

p=0.001
